# Clinical characteristics and risk factors for mortality in pneumonia-associated acute respiratory distress syndrome patients: a single center retrospective cohort study

**DOI:** 10.3389/fcimb.2024.1396088

**Published:** 2024-07-09

**Authors:** Junlu Li, Jiaxin Zhou, Yingshuai Tan, Chunling Hu, Qingshan Meng, Jing Gao, Lihua Xing

**Affiliations:** Department of Respiratory Intensive Care Unit, The First Affiliated Hospital of Zhengzhou University, Zhengzhou, China

**Keywords:** mNGS (metagenomic next-generation sequencing), etiological diagnosis, p-ARDS (pneumonia-associated acute respiratory distress syndrome), clinical characteristics, risk factors for mortality

## Abstract

**Background:**

Pathogenic diversity may have contributed to the high mortality of pneumonia-associated acute respiratory distress syndrome (p-ARDS). Metagenomics next-generation sequencing (mNGS) serves as a valuable diagnostic tool for early pathogen identification. However, its clinical utility in p-ARDS remains understudied. There are still limited researches on the etiology, clinical characteristics and risk factors for 28-day mortality in p-ARDS patients.

**Methods:**

A single center retrospective cohort study of 75 p-ARDS patients was conducted. Patients were categorized into survival and deceased groups based on their 28-day outcomes. A comprehensive clinical evaluation was conducted, including baseline characteristics, laboratory indicators, outcomes and pathogen identification by mNGS and traditional microbiological testing. We then evaluated the diagnostic value of mNGS and identified clinical characteristics and risk factors for 28-day mortality in p-ARDS.

**Result:**

The overall ICU mortality was 26.67%, and the 28-day mortality was 57.33%, with 32 cases (42.67%) in the survival group, and 43 cases (57.33%) in the deceased group. Patients in the deceased group were older than those in the survival group (68(59,73) years vs. 59(44,67) years, *P*=0.04). The average lengths of ICU and hospital stay were 9(5,13) days and 14(7,21) days, respectively. The survival group had longer lengths of ICU and hospital stay (ICU: 11(7,17) days and hospital: 17(9,27) days) compared to the deceased group (ICU: 8(4,11) days and hospital: 12(6,19) days) (*P*<0.05). Survival patients exhibited lower Acute Physiology and Chronic Health Evaluation (APACHE) II score on the 3rd and 7th days, higher lymphocyte counts, higher CD3^+^ and CD8^+^ T cell counts compared to deceased patients (*P*<0.05). Multivariate logistic regression analysis identified age, APACHE II scores on 3rd and 7th days, CD8^+^ T cell count and length of ICU as independent risk factors for 28-day mortality in p-ARDS patients. mNGS demonstrated a significantly higher overall pathogen detection rate (70/75, 93.33%) compared to the traditional method (50/75, 66.67%, *P*=0.022). The average turnaround time (TAT) for mNGS was significantly shorter at 1(1,1) day compared to 4(3,5) days for the traditional method (*P*<0.001).

**Conclusion:**

Metagenome next-generation sequencing can be used as a valuable tool for identifying pathogens in p-ARDS, reducing diagnostic time and improving accuracy. Early application of mNGS alongside traditional methods is recommended for p-ARDS. Furthermore, older age, higher APACHE II scores, lower lymphocyte counts and lymphocyte subset counts were associated with increased mortality in p-ARDS patients, highlighting the importance of timely assessment of immune status and disease severity, especially in elderly.

## 1 Introduction

Acute respiratory distress syndrome (ARDS) manifests as an acute diffuse inflammatory lung injury, instigated by various pathogenic factors, both intra-pulmonary and extra-pulmonary. Its hallmark feature is refractory hypoxemia, rapidly progressing and culminating in multiple organ failure (MODF). ARDS constitutes approximately 10% of admissions to the intensive care unit (ICU), with 23% of ventilated patients affected (Bellani et al., 2016b; Grasselli et al., 2023), and carries a high mortality rate ranging from 30% to 50% (Kang and Kempker, 2019). An initial global literature review indicated that around 33% of hospitalized COVID-19 patients developed ARDS in 2020 (Tzotzos et al., 2020; Bernauer et al., 2023). By June 2020, the COVID-19 pandemic had claimed over 6 million lives worldwide, predominantly due to ARDS (Bos and Ware, 2022; Martin et al., 2022).

ARDS can stem from various triggers, infectious and non-infectious alike, which can inflict direct lung injury via local inflammation or indirect injury mediated by systemic inflammation (Bos and Ware, 2022). Pneumonia, stands as the leading cause of ARDS (Long et al., 2022), constituting a significant global health challenge and ranking among the top 10 causes of death globally (Sun et al., 2020). In recent years, the treatment of pneumonia has become increasingly challenging with the emergence of new pathogenic microorganisms, rising drug-resistant strains, and a growing population of immunosuppressive individuals.

Traditional microbiological diagnostic methods often lead to delayed or inconclusive pathogen identification, hampering precise clinical management, thereby impacting patient outcomes and potentially fostering the spread of unidentified pathogens in vulnerable populations (Gao, 2020). Metagenomic next-generation sequencing (mNGS) technology circumvents the reliance on traditional microbial culture by directly conducting high-throughput sequencing of nucleic acids in clinical samples. It can facilitate rapid and comprehensive detection of various pathogens (including viruses, bacteria, fungi, and parasites) and offer insights into drug resistance patterns. Particularly suited for diagnosing critical and challenging infections (Cao, 2023). This study aims to comprehensively analyze the clinical characteristics and identify risk factors for 28-day mortality in pneumonia-associated ARDS (p-ARDS) patients, concurrently assessing the etiological diagnostic utility of mNGS in p-ARDS.

## 2 Materials and methods

### 2.1 Ethics statement

This study adheres to medical ethics standards and has been reviewed and approved by the Ethical Review Committee of the First Affiliated Hospital of Zhengzhou University (NO: 2021-KY-1000-002). All treatments and procedures were performed with informed consent obtained from the patients’ family members.

### 2.2 Patient recruitment

A retrospective study was conducted at the First Affiliated Hospital of Zhengzhou University, recruiting patients admitted to the Respiratory Intensive Care Unit (RICU) from November 2020 to December 2023. Inclusion criteria were as followed: 1) meeting the Berlin 2012 definition for ARDS diagnosis (ARDS Definition Task Force et al., 2012), 2) pneumonia as the etiology of ARDS, 3) age > 18 years, and 4) complete clinical profiles. Exclusion criteria were as followed: 1) pregnancy, 2) contraindications for fiberoptic bronchoscopy, 3) multiple organ failure, and 4) failure to obtain informed consent when necessary. Patients were categorized into two groups based on their 28-day outcome, survival group and deceased group. A total of 75 patients meeting the inclusion criteria were enrolled. Demographic characteristics, which were documented in electronic medical record system, including age, gender, comorbidity, clinical history, laboratory test indicators, treatment, mNGS findings, and traditional microbiological findings were gathered.

### 2.3 Clinical variables and outcome

Demographic characteristics, including age, gender, comorbidity and time of ARDS onset were recorded.

Laboratory test data collected on the 1st day after enrollment, including arterial blood gas parameters such as PaCO_2_, PaO_2_, and the oxygenation index (PaO_2_/FiO_2_, P/F) on 1, 3, and 7 days. Disease severity was assessed using the Acute Physiology and Chronic Health Evaluation (APACHE) II score and P/F. Routine blood test included white blood cell count (WBC), hemoglobin (Hb), platelet count (PLT), neutrophil count (NE), neutrophil ratio (NE%), lymphocyte count (LY) and lymphocyte ratio (LY%). Renal function indicators, encompassed urine output and creatinine levels, as well as liver function indicators comprised alanine aminotransferase (ALT), aspartate aminotransferase (AST), total bilirubin (Tbil) and albumin levels, were assessed. Inflammatory markers, including procalcitonin (PCT), C-reactive protein (CRP) and erythro sedimentation rate (ESR) were measured. Lymphocyte subset counts, including CD3^+^ T cells, CD4^+^ T cells, and CD8^+^ T cells were determined. Cytokines such as tumor necrosis factor (TNF)-α, interlukin (IL)-6, and IL-10 were also evaluated.

Treatment modalities, including adjunctive measures like invasive mechanical ventilation (IMV), prone position ventilation (PPV) were documented.

Patient outcomes comprised the primary endpoint of 28-day mortality and secondary endpoints including ICU mortality, 90-day mortality, length of ICU, and length of hospital stay (LOS).

### 2.4 Etiology

Within 48 hours of enrollment, bronchoalveolar lavage fluid (BALF) samples were collected from all patients using two sterile containers, each containing 10 ml. These samples were utilized for both traditional microbiological detection and mNGS testing. As not all the detected microorganisms may be pathogenic pathogens of p-ARDS, we determine that whether they were pathogenic or not by combining the patients’ comorbidity, immune status, laboratory test results, imaging manifestations of DR, CT or lung-ultrasound, and response to treatment.

### 2.5 Traditional microbiological detection

Traditional microbiological detection included the methods of special pathological staining, semiquantitative culture and polymerase chain reaction (PCR). Gram staining and weak acid fast staining was used for bacteria identification, acid-fast staining was used for bacteria Mycobacterium tuberculosis complex identification, hexamine silver staining and KOH staining were used for fungi identification, separately. Bacterial were cultured and incubated on blood agar plates, MacConkey agar, and chocolate agar at 35°C for 1-2 days. If typical pathogenic bacterial colonies emerged, further identification and drug sensitivity tests were conducted. Fungi were cultured at 28-30°C for 1-7 days on Sabouraud agar plates and blood agar plates, with continued identification according to fungi colony morphology. Additionally, a high-sensitivity and specificity method of real-time fluorescence PCR was employed to detect human herpesvirus 5 (HHV-5, CMV), aiding in the early diagnosis of CMV infection.

### 2.6 Metagenomics NGS and bioinformatics

Extraction of nucleic acid, preparation of library, and sequencing were conducted as follows. According to the manufacturer’s instructions, DNA of all samples was extracted by the QIAamp^®^ UCP Pathogen DNA Kit (Qiagen). Benzoidase (Qiagen) and Tween20 (Sigma) were used to remove human DNA. Total RNA was extracted by the QIAamp^®^ Viral RNA Kit (Qiagen), and ribosomal RNA was removed by the Ribo Zero rRNA Removal Kit (Illumina). Using the Nextera XT DNA Library Preparation Kit (Illumina, San Diego, CA), cDNA produced by reverse transcriptase and dNTP (Thermo Fisher) were constructed with Libraries of DNA, then Libraries were purified and fragments were screened by magnetic beads. By Qubit dsDNA HS Assay Kit and the High Sensitivity DNA Assay Kit (Agilent), quality of the libraries was assessed on the Agilent 2100 bioanalyzer. Illumina Nextseq CN500 sequencer with 75 single-end sequencing cycles, yielding about 20 million reads per cell load the library pool. Sterile deionized water was prepared as negative controls (NTC) alongside each batch using the same protocol.

Bioinformatics information. High-quality sequencing data were processed by removing low-quality and short reads (< 40bp) reads, and filtering out human host sequences using Burrows-Wheeler Alignment (hg38 and YH sequences). The remaining data, after removing low-complexity reads, were categorized by simultaneous alignment with four microbial genome databases consisting of sort of pathogens, including, viruses, bacteria, fungi, and parasites. From public databases such as NCBI, EBI, or GenBank, the categorized databases were downloaded and optimized. Then a large number of parameters for pathogen identification were calculated and extracted from the microbial gene database information, and the etiology results of mNGS were finally obtained through the interpretation of clinical microbiology experts.

### 2.7 Statistical analysis

Data statistical analysis was performed using IBM SPSS Statistics 21.0 (IBM, NY, USA). Normality tests were conducted on measurement data. Normally distributed measurement data were represented as mean (M) ± standard deviation (SD), and independent sample t-tests were used for inter-group comparison. Non normally distributed measurement data were represented by median and interquartile intervals, median (interquartile range [IQR]), and Wilcoxon Mann-Whitney tests were used for inter-group comparison. Enumeration data were expressed as frequency and rate, and inter group comparison were performed using chi-square tests. Variables with statistically significant differences in the results of univariate analysis were included in multivariate logistic regression analysis to identify independent risk factors for 28-day mortality. A supplementary analysis of the Variance Inflation Factor (VIF) to identify the correlation between variables in multiple regression model. By Log rank test, Kaplan-Meier curves were generated to compare 28-day survival rates among different variables. The area under the curve (AUC) of the receiver operating characteristic (ROC) was calculated to evaluate the effectiveness of each parameter and the joint predictive model. All tests were conducted using a bilateral test, with α=0.05 was used as the significance level.

## 3 Results

**Figure 1:**
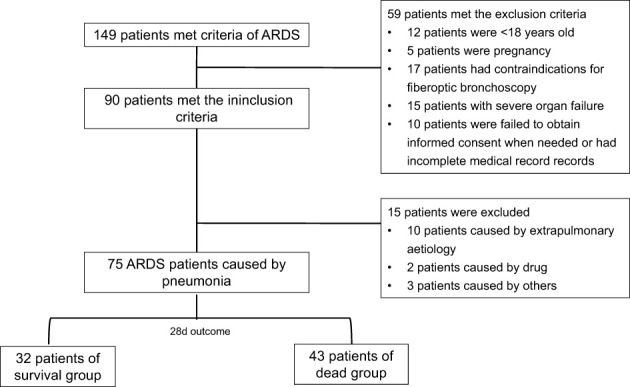
Flowchart of Patient recruitment.

Of the 149 cases, 75 with a diagnosis of p-ARDS were extracted from our database (**Figure 1**).

### 3.1 Clinical variables and outcome

#### 3.1.1 Demographic characteristics

75 patients participated in the study, comprising 45 males and 30 females, with an average age of 66 (51, 70) years. Among them, 32 patients (42.67%) were categorized in the survival group, while 43 patients (57.33%) were in the deceased group. The average age of patients in the deceased group was 68(59,73) years, which was older than those in the survival group with 59(44,67) years, showing a statistical difference (*P*=0.004).The predominant comorbidity among the p-ARDS population was immunosuppressive diseases, including autoimmune diseases, hematologic diseases, neoplastic diseases, and organ transplantation, accounting for 52.0%. Other common comorbidities in the p-ARDS population included cardiovascular disease (32/75, 42.67%), diabetes (12/75, 16.0%), chronic lung diseases (11/75, 14.67%), cerebrovascular disease (11/75, 14.67%) and chronic kidney disease (9/75, 12.0%), with no statistical differences between the two groups (*P*>0.05). The prevalence of smoking and drinking showed no statistical differences between the survival and deceased groups (history of smoking, 20(62.5) vs. 29(67.4), *P*=0.656; history of drinking, 25(78.1) vs. 35(81.4), *P*=0.726). The onset time of ARDS in the survival group (72 [48, 120] days) was similar to that in the deceased group (72 [24, 144] days). The IMV duration in the survival group (0(0,156) hours) was significantly shorter than deceased ones (99(21,231) hours, *P*<0.001). While application ratio of the adjunctive treatment, PPV, was similar in the two group (5(16.67%) vs. 13(30.23%), *P*=0.186) (**Table 1**).

**Table 1:** Patients’ demographic characteristics.

	Total patients (N = 75)	Survival group (N = 32)	Deceased group (N = 43)	*P* value
**Age (years)**	66 (51,70)	59 (44,67)	68 (59,73)	0.004
**Sex n (%)**				0.567
Male	45 (60)	18 (56.3)	27 (62.8)	
Female	30 (40)	14 (43.8)	16 (37.2)	
Comorbidities (%)				
Immunosuppressive diseases	39 (52)	40.6	60.5	0.089
Chronic lung diseases	11 (14.67)	9.4	18.6	0.431
Cardiovascular disease	32 (42.67)	34.4	48.8	0.210
Diabetes	12 (16)	9.4	20.9	0.177
Cerebrovascular disease	11 (14.67)	12.5	16.3	0.898
Chronic kidney disease	9 (12)	12.5	11.6	0.908
Smoking n (%)				0.656
Never	49 (65.33)	12 (37.5)	14 (32.6)	
Former/Current	26 (34.67)	20 (62.5)	29 (67.4)	
Alcohol use n (%)				0.726
Never	60 (80.0)	7 (21.9)	8 (18.6)	
Former/Current	15 (20.0)	25 (78.1)	35 (81.4)	
**ARDS onset time (h)**	72 (48,120)	72 (48,120)	72 (24,144)	0.850
Disease severity at admission				
APACHE II score,	17 (13,21)	16 (10,21)	18 (14,22)	0.064
PaO_2_/FiO_2_ (mmHg)	112.22 (76.00,157.31)	151.17 (84.62,190.27)	101.60 (67.28,144.63)	0.007
**Severity of ARDS, n (%)**				0.227
Mild	8 (10.67)	5 (15.62)	3 (6.98)	
Moderate	36 (48)	17 (53.12)	19 (44.19)	
Severe	31 (41.33)	10 (31.25)	21 (48.84)	
**IMV duration (h)**	72 (0,216)	0 (0,156)	99 (21,231)	<0.001
**PPV (n%)**	18 (24.66)	5 (16.67)	13 (30.23)	0.186

Immunosuppressive diseases, encompassing autoimmune diseases, hematologic diseases, neoplastic conditions and post-organ transplantation states; Chronic lung diseases, include chronic obstructive pulmonary disease (COPD), bronchial asthma, chronic pulmonary embolism, interstitial lung disease, bronchiectasis, lung abscess and postoperative lung cancer; Cardiovascular disease, comprises coronary atherosclerotic heart disease, hypertension, chronic heart failure and arrhythmia; Cerebrovascular disease, compassing cerebral hemorrhage, newly diagnosed cerebral infarctions, and postoperative brain injuries.

ARDS, acute respiratory distress syndrome.

ARDS onset time, was identified as the length of a known clinical insult or new/worsening respiratory symptoms developed to ARDS.

APACHE II score, acute physiology and chronic health evaluation II score.

IMV, invasive mechanical ventilation.

PPV, prone position ventilation.

#### 3.1.2 Laboratory data

Although the APACHE II scores and P/F ratio on the 1st day did not differ between the groups, the deceased group exhibited higher APACHE II scores on the 3rd and 7th days compared to the survival group (3rd day, 20(15,23) vs. 20(15,23), *P*<0.001; 7th day, 19(13,23) vs. 10(6,15), *P*<0.001). Additionally, the deceased group had lower P/F ratios (3rd day, 101.52(68.99,147.66) vs. 124.76(102.15,237.03), *P*=0.033; 7th day, 111.81(73.55,155.44) vs. 178.33(131.58,248.92), *P*=0.001). The lymphocyte count in deceased group was 0.41(0.22,0.82)×10^9^/L, which was lower than that in the survival group 0.70(0.46,1.03)×10^9^/L, with a significant difference (*P*=0.018). Meanwhile, not only the total number of lymphocyte subsets CD3^+^ T cell of deceased patients were slightly lower than in the survival group (293.47(136.07,598.98)/uL vs. 562.36(302.42,926.10)/uL, *P*=0.019), but also CD8^+^ T cell counts were lower in deceased group compared to the survival group (138.00(66.32,208.84)/uL vs. 247.93(134.65,356.00)/uL, *P*=0.005). However, no significant differences were observed in routine blood tests, renal function, liver function, inflammatory markers, and cytokine levels between the two groups (**Table 2**).

**Table 2:** Patients’ laboratory data.

		Survival group (N = 32)	Deceased group (N = 43)	*P* value
Disease assessment				
**1d**	P/F (mmHg)	151.17 (84.62,190.27)	101.60 (67.28,144.63)	0.007
**3d**	P/F (mmHg)	124.76 (102.15,237.03)	101.52 (68.99,147.66)	0.033
**7d**	P/F (mmHg)	178.33 (131.58,248.92)	111.81 (73.55,155.44)	0.001
**1d**	APACHE II score	16 (10,21)	18 (14,22)	0.064
**3d**	APACHE II score	13 (9,17)	20 (15,23)	<0.001
**7d**	APACHE II score	10 (6,15)	19 (13,23)	<0.001
Lab test				
**Routine blood test**	WBC (×10^9^/L)	11.48 ± 8.12	10.46 ± 5.96	0.801
	HB (g/L)	110.39 ± 23.87	103.75 ± 25.07	0.235
	PLT (×10^9^/L)	187.72 ± 84.43	174.04 ± 107.79	0.993
	Neutrophil count (×10^9^/L)	8.38 (4.38,13.27)	9.20 (5.05,13.70)	0.510
	Neutrophil ratio (%)	90.55 (77.95,92.70)	92.90 (82.30,95.80)	0.069
	Lymphocyte count (×10^9^/L)	0.70 (0.46,1.03)	0.41 (0.22,0.82)	0.018
	Lymphocyte ratio (%)	6.40 (4.28,15.03)	5.20 (2.10,8.50)	0.057
**Renal function**	urine output (mL/d)	1050 (438,1250)	850 (400,1775)	0.777
	Creatinine (ummol/L)	67.00 (49.50,102.50)	72.00 (51.30,138.23)	0.559
**Liver function**	ALT (U/L)	24.00 (14.00,45.00)	22.00 (13.75,39.75)	0.517
	AST (U/L)	41.50 (23.25,47.00)	34.00 (19.00,50.25)	0.810
	TBIL (ummol/L)	11.20 (5.70,18.90)	9.20 (6.90,15.75)	0.851
	Albumin (g/L)	30.60 (27.10,32.80)	29.00 (25.90,34.10)	0.630
**Inflammatory index**	PCT (ng/mL)	0.84 (0.35,8.53)	0.36 (0.15,1.48)	0.060
	CRP (mg/L)	131.87 (61.14,214.75)	111.33 (60.79,195.50)	0.315
	ESR (mm/h)	63.00 (42.00,85.00)	33.00 (22.00,82.00)	0.087
**Lymphocyte subsets count**	CD3^+^ T cell (/uL)	562.36 (302.42,926.10)	293.47 (136.07,598.98)	0.019
	CD4^+^ T cell (/uL)	228.00 (145.86,393.79)	114.15 (56.90,303.62)	0.084
	CD8^+^ T cell (/uL)	247.93 (134.65,356.00)	138.00 (66.32,208.84)	0.005
**Cytokine**	TNF-α (pg/mL)	1.91 (1.26,3.27)	2.14 (1.44,2.64)	0.775
	IL-6 (pg/mL)	24.30 (6.16,213.28)	42.33 (8.92,106.20)	0.720
	IL-10 (pg/mL)	2.66 (2.16,4.33)	2.86 (2.17,6.66)	0.508

1d, 1st day; 3d, 3rd day; 7d, 7th day; P/F, oxygenation index (PaO_2_/FiO_2_); APACHE II score, acute physiology and chronic health evaluation II score; WBC, white blood cell count; Hb, hemoglobin concentration; PLT, platelet count; NE, neutrophil count; NE%, neutrophil ratio; LY, lymphocyte count; LY%, lymphocyte ratio; ALT, alanine aminotransferase; AST, aspartate aminotransferase; Tbil, total bilirubin; PCT, procalcitonin; CRP, C-reactive protein; ESR, erythro sedimentation rate; IL, interlukin; TNF, tumor necrosis factor.

These above findings suggested a close relationship between immune status and prognosis. So we made an extra comparison. Patients were divided into immunosuppressive group (N=39) and immunocompetent group (N=36), according to the underlying disease and medication history (Ramirez et al., 2020). The comparison of laboratory data showed that immunosuppressive p-ARDS patients had higher APACHE II scores on the 3rd and 7th days, lower WBC count, lower PLT count, lower Neutrophil count, lower TBIL and lower CD4^+^ T cell count than the immunocompetent ones (**Supplementary Table 1** in the supplement).

#### 3.1.3 Patients’ outcome

There were statistically significant differences in both ICU mortality and 90-day mortality between the two groups (survival group vs deceased group, ICU mortality, 0% vs. 18.6%, *P*=0.010; 90d mortality, 0% vs. 100.0%, *P<*0.001). The average length of ICU was 9(5,13) days and the average LOS was 14(7,21) days. The length of ICU in the survival group was 11 (7, 17) days, which was significantly longer than in the deceased group (8(4, 11) days, *P*=0.018). However, there was no statistically significant difference in LOS between the two groups (survival group: 17(9, 27) days vs. dead group: 12(6, 19) days, *P*=0.067) (**Table 3**).

**Table 3:** Patients’ outcome.

		Survival group (N = 32)	Deceased group (N = 43)	*P* value
**Primary endpoint**	28-day mortality (%)	0	57.3	<0.001
**Secondary endpoint**	ICU mortality (%)	0	18.6	0.010
	90d mortality (%)	0	100	<0.001
	Length of ICU (d)	11 (7,17)	8 (4,11)	0.018
	Length of hospital stay (LOS, d)	17 (9,27)	12 (6,19)	0.067

The immunosuppressive group had higher mortality of ICU, 28-day and 90-day than immunocompetent group (immunosuppressive group vs immunocompetent group, ICU mortality, 15.38% vs. 5.56%, *P*=0.316; 28-day mortality, 66.67% vs. 47.22%, *P=*0.089; 90-day mortality, 71.79% vs. 50%, *P=*0.053), but without significant differences (**Supplementary Table 2** in the supplement).

Moreover, as P/F is the widely recognized indicators for mortality prediction, we conducted the comparison between the different severity groups. The mild ARDS gourp (P/F 200~300mmHg), including 8 patients, had the lowest mortality both of ICU, 28-day and 90-day [ICU, 0(0.00%), 28-day, 3(37.5%), 90-day, 3(37.5%)]. while severe ARDS gourp (P/F ~100mmHg), including 31 patients, had the highest mortality both of ICU, 28-day and 90-day (ICU, 3(9.7%), 28-day, 21(67.7%), 90-day, 22(71.0%)], but there were not statistically significant differences in mortality in the three groups (**Supplementary Table 3** in the supplement).

**Figure 2:**
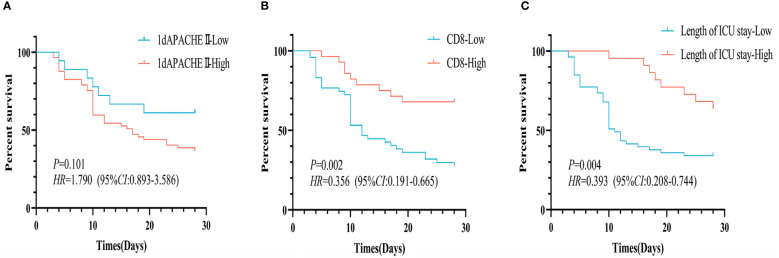
Kaplan-Meier survival curves for 28-day survival of p-ARDS patients with different indexes. **(A)** No significant differences in survival rate between the patients with different APACHE II score on 1st day (P=0.101). **(B)** The patients with averaged CD8^+^ T cell count ≥ 220/uL exhibited a higher survival rate than those with counts < 220/uL (P=0.002). **(C)** The patients with a length of ICU ≥ 13 days had a higher survival rate than those with below 13 days (P=0.004).

Then, according to APACHE II score on 1st day (cut-off value, 13), CD8^+^ T cell (cut-off value, 220/uL), and length of ICU (cut-off value, 13 days), Kaplan-Meier survival curves were performed (**Figure 2**). No significant differences in cumulative survival were observed between the patients with APACHE II score on 1st day (*P*=0.101, **Figure 2A**). But the patients with averaged CD8^+^ T cell count ≥ 220/uL exhibited a higher cumulative survival for 28-day mortality compared to those with counts below 220/uL (*P*=0.002, **Figure 2B**). Additionally, patients with a length of ICU exceeding 13 days demonstrated higher cumulative survival than those with below 13 days (*P*=0.004, **Figure 2C**).

**Figure 3:**
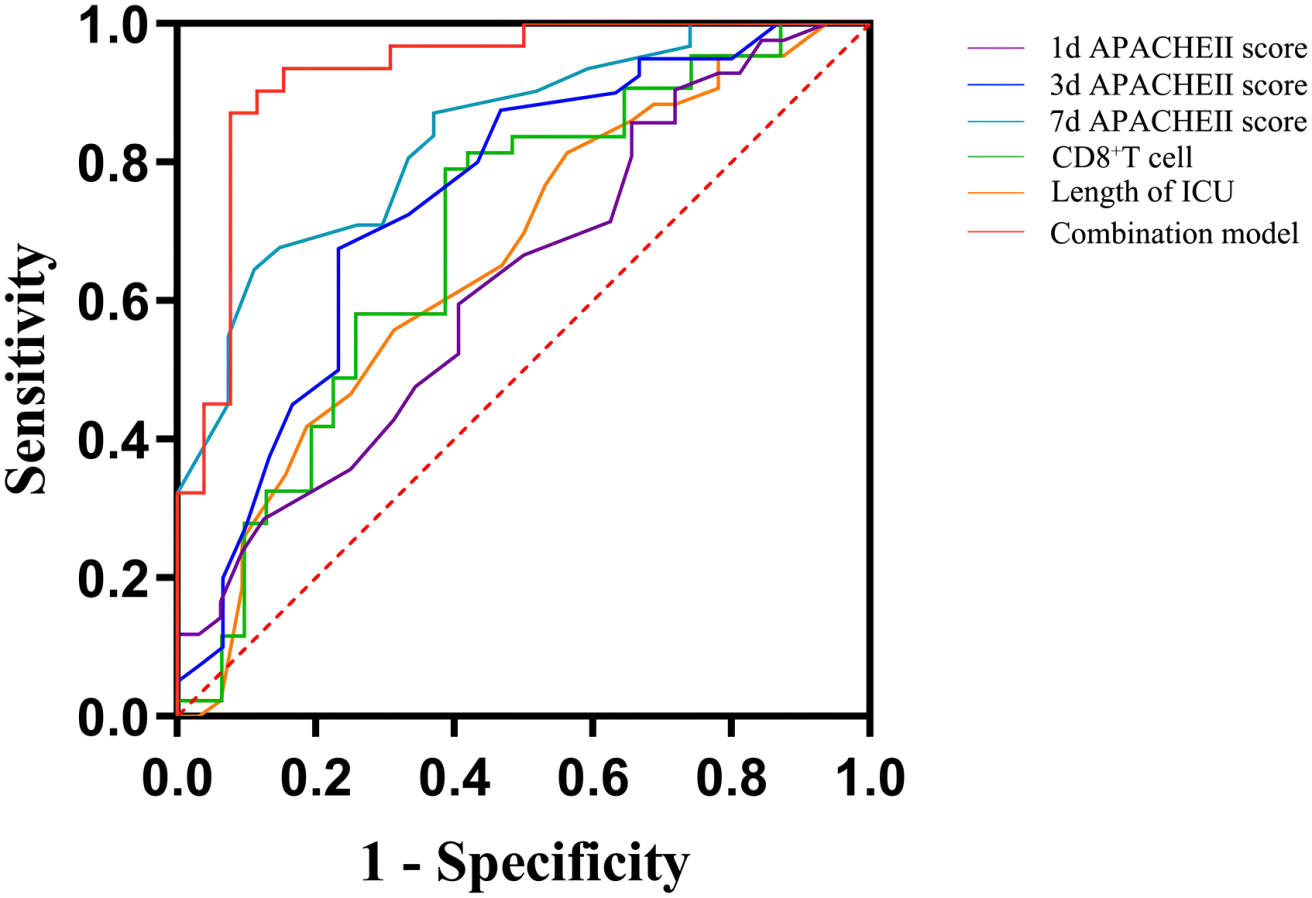
Receiver operating curves (ROC) for predicting 28-day survival of p-ARDS with different related variables. 1d, 1st day; 3d, 3rd day; 7d, 7th day; APACHE II score, acute physiology and chronic health evaluation II score.

In the population of patients with p-ARDS, our analysis revealed that age, APACHE II score on the 3rd and 7th day and CD8^+^ T cell count as independent risk factors for 28-day mortality in p-ARDS patients, as determined by multivariate logistic regression analysis (**Table 4**). Considering the potential interactions between variables in multiple regression models, we conducted a supplementary analysis of the Variance Inflation Factor (VIF). The analysis showed that the VIF values of all 8 models were less than 5, indicating that there was no significant correlation between the included variables. Then, the effectiveness of each parameter was evaluated using the area under the curve (AUC) of the receiver operating characteristic (ROC) curve. Subsequently, a comprehensive risk prediction model was constructed, incorporating variables such as age, APACHE II scores on days 1, 3, and 7, CD8^+^ T cell count and length of ICU. This model exhibited a sensitivity of 87.1%, specificity of 92.3%, and an AUC of 0.928 (**Figure 3**; **Tables 4**, **5**).

**Table 4:** Association between risk factors and 28-day mortality risk.

	MODEL 1		MODEL 2	
	OR (95% CI)	*P* value	OR (95% CI)	*P* value
**1d APACHE II score**	1.082 (1.007,1.163)	0.031	1.083 (1.003,1.169)	0.041
**3d APACHE II score**	1.163 (1.059,1.278)	0.002	1.149 (1.046,1.263)	0.004
**7d APACHE II score**	1.268 (1.118,1.437)	<0.001	1.251 (1.103,1.419)	<0.001
**Lymphocyte count (**×**10^9^/L)**	0.815 (0.505,1.317)	0.404	0.818 (0.511,1.309)	0.401
**CD3^+^ T cell (/uL)**	0.999 (0.997,1.000)	0.036	0.999 (0.998,1.000)	0.110
**CD8^+^ T cell (/uL)**	0.994 (0.990,0.998)	0.005	0.994 (0.989,0.998)	0.008
**Length of ICU (d)**	0.918 (0.853,0.988)	0.022	0.916 (0.849,0.988)	0.023

Model 1 represents unadjusted confounding factors, while Model 2 represents adjusted for age, history of smoking and drinking. And a supplementary analysis of the Variance Inflation Factor (VIF) was conducted. The analysis showed that the VIF values of all 8 models were less than 5, indicating that there was no significant correlation between the included variables.

1d, 1st day; 3d, 3rd day; 7d, 7th day; APACHE II score, acute physiology and chronic health evaluation II score.

**Table 5:** Predictive analysis of the 28-day outcome of p-ARDS patients.

Variables	AUC	95%CI	sensitivity	specificity	*P* value	cutoff value
**1d APACHE II score**	0.626	0.498~0.754	0.857	0.344	0.064	12.5
**3d APACHE II score**	0.747	0.627~0.866	0.675	0.767	<0.001	16.5
**7d APACHE II score**	0.837	0.736~0.938	0.645	0.889	<0.001	16.5
**CD8^+^ T cell (/uL)**	0.692	0.566~0.819	0.7907	0.6129	0.005	220.1
**Length of ICU (d)**	0.660	0.533~0.786	0.814	0.438	0.019	12.5
**combination model**	0.928	0.856~1.000	0.871	0.923	<0.001	–

Note: The combination model included the variables, age, APACHE II score on days 1, 3 and 7, CD8^+^ T cell count and length of ICU.

1d, 1st day; 3d, 3rd day; 7d, 7th day; APACHE II score, acute physiology and chronic health evaluation II score.

### 3.2 Etiology

**Figure 4:**
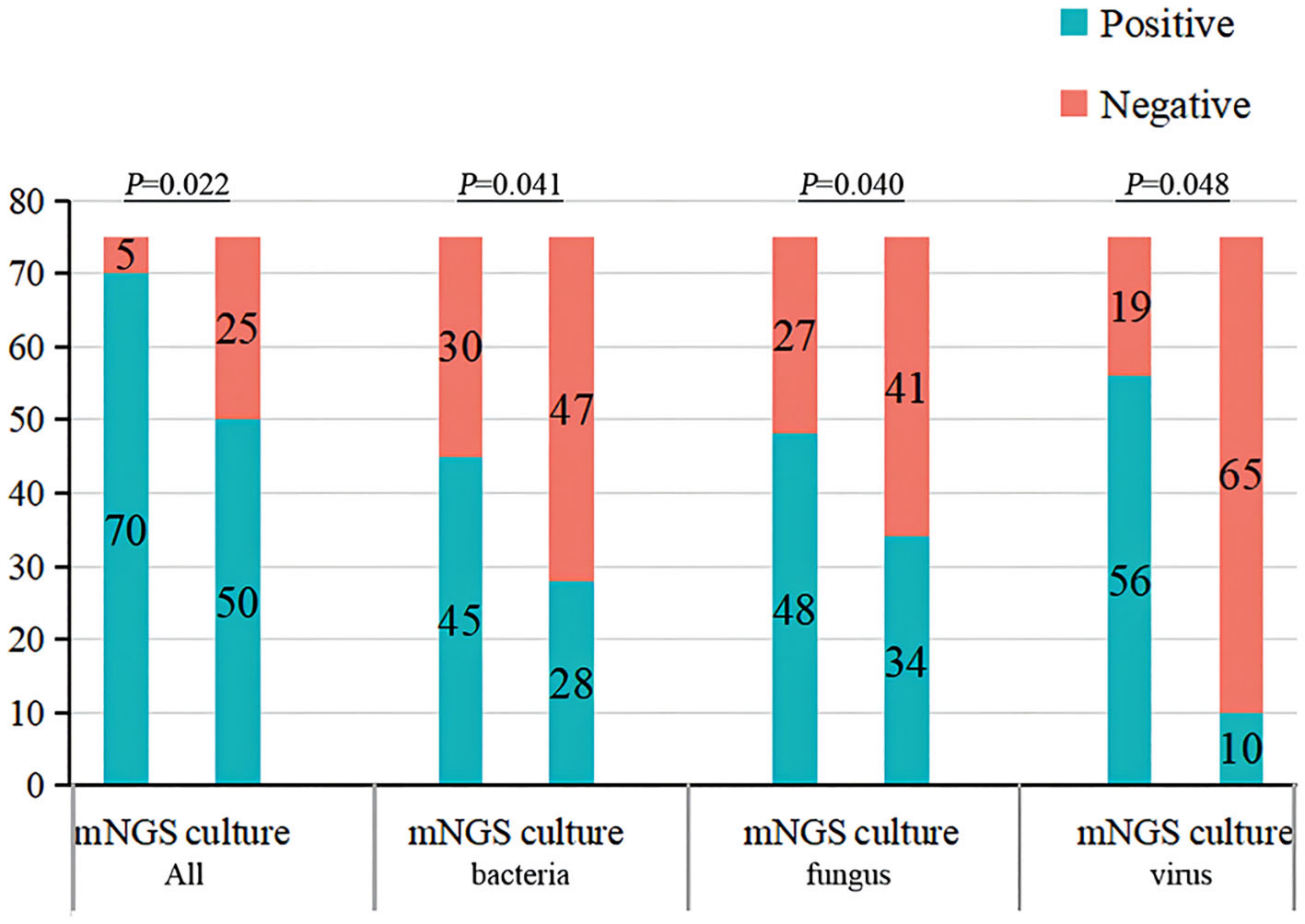
The overall detection rate and different pathogens detection rate of traditional microbiological methods and mNGS. mNGS, metagenomics next-generation sequencing.

**Figure 5:**
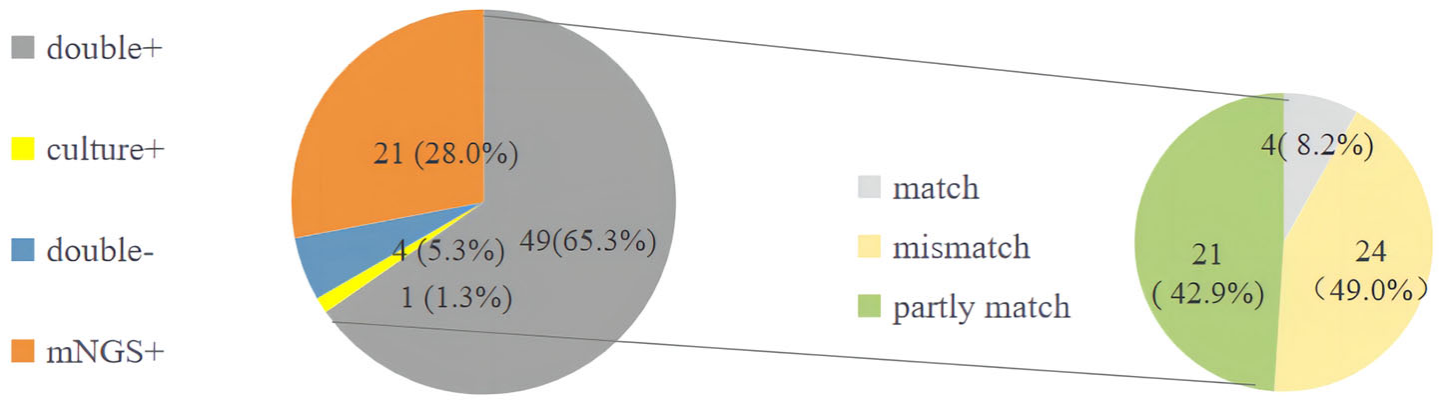
The detection of pathogens of traditional microbiological methods and mNGS. mNGS, metagenomics next-generation sequencing.

Compared with traditional microbiological methods, the overall positive detection rate of pathogens was significantly higher with metagenomics next-generation sequencing (mNGS) (70/75, 93.33% vs. 50/75, 66.67%, *P*=0.022). Additionally, the average turnaround time (TAT) of mNGS was notably shorter at 1(1,1) day, compared to traditional methods, which had a TAT of 4(3,5) days (*P*<0.001) (**Figures 4**, **5**; **Table 6**).

**Table 6:** Patients’ etiology.

		Total patients (N = 75)	Survival group (N = 32)	Deceased group (N = 43)	*P* value
**Positive rate n, (%)**	mNGS	70 (93.3)	29 (90.6)	41 (95.3)	0.417
	Traditional test	50 (66.7)	21 (65.6)	29 (67.4)	0.869
**Turnaround time (d)**	mNGS	1 (1,1)	1 (1,1)	1 (1,1)	0.787
	Traditional test	4 (3.5)	5 (3,5)	4 (3,5)	0.179
**Overall positive rate n, (%)**	Bacteria	52 (69.3)	18 (56.3)	34 (79.1)	0.034
	Fungi	56 (74.7)	20 (62.5)	36 (83.7)	0.037
	Viral	56 (74.7)	20 (62.5)	36 (83.7)	0.037
**Overall Type of pathogen n, (%)**	none	4 (5.3)	3 (9.4)	1 (2.3)	0.003
	One type	9 (12.0)	6 (18.8)	3 (7.0)	
	Two types	31 (41.3)	17 (53.1)	14 (32.6)	
	≥Three types	31 (41.3)	6 (18.8)	25 (58.1)	

mNGS, metagenomics next-generation sequencing.

Traditional test, traditional microbiological methods of pathogenic detection.

Although there was no statistically significant difference in the positive detection rate between mNGS and traditional methods within either the survival group or the deceased group, the overall positive detection rates using mNGS plus traditional methods were significantly different between the survival and deceased groups. Specifically, compared to the survival group, the deceased group exhibited higher positive detection rates of bacteria, fungi, and viruses, and also had a higher rate of detecting more than two types of pathogens (**Figures 4**, **5**; **Table 6**).

**Figure 6:**
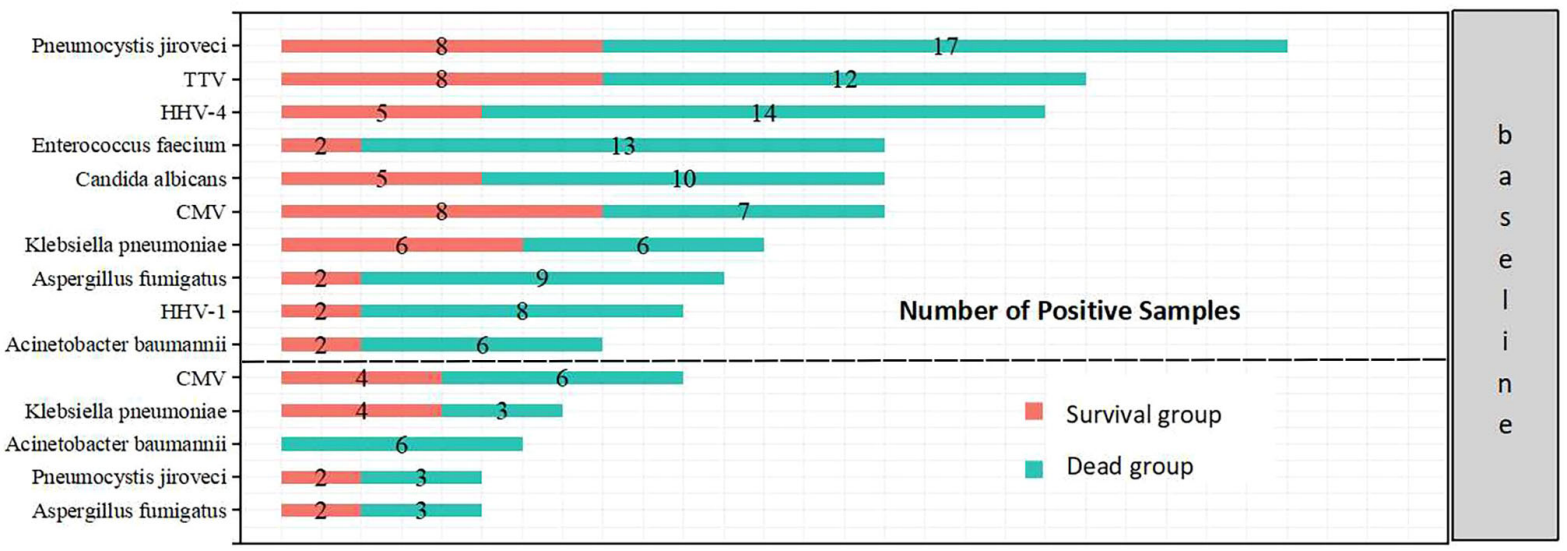
Top 10 pathogens detected by mNGS (above) and top 5 pathogens detected by traditional microbiological methods (below) in the two groups.

The overall positive pathogen detection rate in deceased group was higher than that in survival group, with 79.1% of cases indicating bacterial infection, 83.7% indicating fungal infection, and 83.7% indicating viral infection, compared to the survival group (56.3% bacterial infection, 62.5% fungal infection, and 62.5% viral infection, *P*<0.05). Furthermore, the occurrence of mixed infections with more than two pathogens in the deceased group (58.1%) was higher than that in the survival group (18.8%). The top 10 prevalent pathogens identified by mNGS included Pneumocystis jiroveci, Torque teno virus (TTV), human herpesvirus 4 (HHV-4), Enterococcus faecium, Candida albicans, CMV, Klebsiella pneumoniae, Aspergillus fumigatus, HHV-1 and Acinetobacter baumannii. These pathogens differed from those identified by traditional methods, which included CMV, Klebsiella pneumoniae, Acinetobacter baumannii, Pneumocystis jiroveci, and Aspergillus fumigatus (**Figures 5**, **6**).

## 4 Discussion

In this retrospective study, we enrolled a total of 75 pulmonary-associated acute respiratory distress syndrome (p-ARDS) patients and objected to assess the etiological diagnostic value of metagenomics next-generation sequencing (mNGS) compared to the traditional microbiological methods. We found mNGS had not only higher positive detection rate, but also shorter turnaround time than traditional method. Subsequently we identified the clinical characteristics and risk factors for 28-day mortality. Results showed that independent risk factors for 28-day mortality in p-ARDS patients included age, APACHE II score on 3rd and 7th days, CD8^+^ T cell counts and length of ICU.

The average age of deceased p-ARDS patients was 68 years, which was significantly older than the survival ones. This difference prognosis may be attributed to the presence of multiple comorbidities and poor functional status among elderly patients (Patel et al., 2024; Wu et al., 2024). Consistent with findings from the LUNGSAFE study, older ARDS patients with a mean age of 61 years, had a higher in-hospital mortality rate than those below 61 years (Bellani et al., 2016a). Recent studies also suggested that age is independently associated with increased mortality in ARDS patients (Song et al., 2020; Sehgal et al., 2021). So, we should pay more attention to the elderly ARDS patients.

In addition to age, immune status also played an important role in prognosis. The prevalence of immunosuppressive diseases, including autoimmune diseases, hematologic diseases, neoplastic diseases and post-organ transplantation, was higher in the deceased p-ARDS patients. Their lymphocyte count and lymphocyte subsets (CD3^+^ T and CD8^+^ T cells) counts were significantly lower than survival ones. The immunosuppressive p-ARDS patients had higher APACHE II score, lower WBC count, PLT count, neutrophil count and CD4^+^ T cell count, as well as higher mortality than immunocompetent ones. These findings suggest a close relationship between immune status and prognosis. which was consistent with previous reports. Existing literature showed that immunosuppressive patients are more susceptible to ARDS, with greater severity, less responsiveness to various treatments, and higher mortality compared to immunocompetent patients (Azoulay et al., 2018; Cheng et al., 2022).

Interestingly, while most of previous studies have emphasized the crucial role of CD4^+^ T cells in immune status (Freiwald et al., 2020; Wik and Skålhegg, 2022), our study revealed no significant difference in CD4^+^ T cell counts between survival and deceased patients with p-ARDS. Conversely, we found significant differences in CD8^+^ T cell counts. Multivariate logistic regression analysis and Kaplan-Meier curves also demonstrated a close association between low CD8^+^ T cell counts and 28-day mortality outcomes in p-ARDS patients. This result may be related to the function of CD8^+^ T cell. CD8^+^ T cells, as adaptive immune killer cells, play a crucial role in eliminating infected cells and tumor cells. A decrease in peripheral blood CD8^+^ T cell counts may indicate migration to infected sites, contributing to inflammatory reactions and reflecting worsening patient condition (Li et al., 2020; Wang et al., 2020; Bergamaschi et al., 2021). Similar research can be found in sepsis patients, where reductions in peripheral blood CD8^+^ T cell count were observed in sepsis patients with deteriorating conditions (Tang et al., 2023).

We often think that patients with mild illness have a shorter hospital stay than those with severe illness. But our statistical analysis revealed that the deceased p-ARDS had a shorter length of ICU compared to the survival patients, which was different from conventional cognition. This observation may be attributed to the high mortality within 7~14 days in ARDS, especially in severe ARDS patients. Rapid exacerbation seen in the deceased group, leading to insufficient treatment time, poor outcomes and short LOS. Similar results in the SOAP study, which included 393 ARDS patients, revealed severe ARDS had shorter length of ICU and length of hospital stay than less severe ARDS patients (Vincent et al., 2010). The longer length of ICU observed in surviving p-ARDS patients in our study may be multifactorial, possibly related to an increased rate of more complications, more adjunctive treatments or hospital-acquired infections, as suggested by some studies (Vincent et al., 2010; Grasselli et al., 2019). But to confirmed the multi-factors need further researches.

Although we have made comprehensive treatment and advanced life support treatment, the mortality of p-ARDS patients is still high. The overall ICU mortality rate of ARDS was 18.6%, consistent with current literature data (Bos and Ware, 2022). However, the 28-day mortality rate was as high as 57.33%, much higher than ICU mortality, which may be attributed to several factors. Firstly, some patients who survived in the ICU may have opted to discontinue hospitalization due to economic or family reasons, resulting in subsequent mortality after discharge. Secondly, literature indicates that the long-term quality of life and survival rates of ARDS patients are significantly decreased, leading to a much higher 28-day mortality rate compared to the ICU mortality rate (Combes et al., 2018). In addition, as the severity of the condition worsens, the mortality rate of both ICU, 28-day and 90-day in ARDS patients significantly increases. Therefore, detection of the pathogenic factors of p-ARDS as early as possible and timely control of ARDS progression, are crucial to improve patients’ prognosis.

High mortality of p-ARDS can be attributed to multiple-pathogenic infections, including rare and emerging microorganisms, particularly in immunosuppressive hosts. In our study, we found deceased p-ARDS patients have high the overall detection and occurrence of mixed infections with >2 pathogens. As their critical condition, prolonged mechanical ventilation and numerous invasive life support interventions such as ECMO were often necessitated, which significantly increased the risk of polymicrobial infections and drug-resistant bacterial infections. So, mNGS was used as a promising tool for its rapid turnaround time and high sensitivity in p-ARDS, especially for rare, novel, and complicated infections (Shi et al., 2022). Common in-hospital pathogens such as Klebsiella pneumoniae, Acinetobacter baumannii and Enterococcus faecium with high rate of multi-drug-resistance were most prevalent in p-ARDS. Pathogens with historically low incidence rates, such as Candida albicans, CMV and Aspergillus fumigatus were also extensively detected, possibly due to the immunosuppressive status and invasive treatments of p-ARDS patients. Rare pathogens like Pneumocystis jiroveci were mostly detected by mNGS, primarily infected hosts with immunosuppression. Pathogens with lower pathogenicity, such as Torque teno virus (TTV) and HHV-4, were also evaluated in our study, with many being involved in mixed infections with other pathogens. They may also reflected the patients’ impaired immune status and severe condition. Although mNGS has the high detection rate of pathogens, it also has the false negative outcome, like the traditional method. The highest negative predictive value of mNGS is 97.89%, but not 100% (Qian et al., 2022). In our study, we found few cases of double negative detected pathogen. We still diagnosed them with p-ARDS, combining their underlying disease, immune status, laboratory test results, imaging manifestations of DR, CT or lung-ultrasound, and response to antimicrobial therapy. So we should make a comprehensive diagnosis in pathogens according to clinic, not only rely on the mNGS.

There are several limitations to our study, notably the small, enrolled sample size of 75 patients. It’s essential to validate the diagnostic efficacy of mNGS for both pathogenic and background pathogens with larger sample sizes to draw more reliable conclusions. The data collected primarily at the time of patient enrollment lacks trends in data changes during treatment, thereby limiting our ability to assess changes in patient condition.

## 5 Conclusion

Metagenomic next-generation sequencing (mNGS) proves to be a valuable tool in determining the etiology of p-ARDS. The turnaround time of mNGS has been confirmed to be faster than traditional method, which is beneficial in the timely diagnosis of infections. Another important advantage of mNGS is its capability to detect pathogens in mixed infections and drug-resistance within a short time frame, a challenge for traditional microbiological detection. This direct typing enables immediate initiation of targeted treatment and optimized treatment process for infections. Therefore, early application of mNGS is recommended for severe p-ARDS patients as early as possible. Meanwhile, older age, higher APACHE II score, lower CD8^+^ lymphocyte count and shorter length of ICU were associated with a greater risk of mortality in p-ARDS patients, highlighting the need for increased attention to elderly patients, immune status and timely assessment of patients’ condition.

## Data availability statement

The datasets presented in this study can be found in online repositories. The names of the repository/repositories and accession number(s) can be found below: https://www.ebi.ac.uk/ena, PRJEB73552.

## Ethics statement

The studies involving humans were approved by ethical review committee of the First Affiliated Hospital of Zhengzhou University. The studies were conducted in accordance with the local legislation and institutional requirements. The participants provided their written informed consent to participate in this study. Written informed consent was obtained from the individual(s) for the publication of any potentially identifiable images or data included in this article.

## Author contributions

JL: Data curation, Formal analysis, Funding acquisition, Investigation, Methodology, Project administration, Resources, Software, Writing – original draft, Writing – review & editing. JZ: Data curation, Methodology, Resources, Writing – original draft. YT: Data curation, Methodology, Resources, Writing – original draft. CH: Methodology, Supervision, Validation, Writing – review & editing. QM: Data curation, Methodology, Supervision, Visualization, Writing – review & editing. JG: Conceptualization, Funding acquisition, Methodology, Resources, Supervision, Validation, Writing – review & editing. LX: Conceptualization, Funding acquisition, Resources, Supervision, Validation, Writing – review & editing. 
