# Supplementary material for: Clinical characteristics and risk factors for mortality in pneumonia-associated acute respiratory distress syndrome patients: a single center retrospective cohort study
Source: Front Cell Infect Microbiol. 2024 Jul 9;14:1396088. doi: 10.3389/fcimb.2024.1396088 (PMC11263095; doi:10.3389/fcimb.2024.1396088)
Supplement: Supplementary file 1 [file Table_1.docx]

eTABLE 1 Patients’ laboratory data

|  | | **Immunocompetent group**  **(N=39)** | **Immunosuppressive group**  **(N=36)** | ***P* value** |
| --- | --- | --- | --- | --- |
| **Disease assessment** | |  |  |  |
| 1d | P/F (mmHg) | 111.36 (79.07, 165.08) | 132.57 (71.42, 149.50) | 0.596 |
| 3d | P/F (mmHg) | 120.45 (73.00, 218.33) | 115.00 (75.58, 145.03) | 0.479 |
| 7d | P/F (mmHg) | 137.88 (94.60, 195.17) | 133.71 (79.40, 189.96) | 0.941 |
| 1d | APACHE Ⅱ score | 15.00 (12.75, 21.00) | 18.00 (14.25, 21.00) | 0.306 |
| 3d | APACHE Ⅱ score | 15.00 (12.00, 20.00) | 19.00 (15.00, 22.00) | 0.030 |
| 7d | APACHE Ⅱ score | 10.00 (8.00, 16.50) | 17.00 (12.00, 21.25) | 0.010 |
| Lab test | |  |  |  |
| Routine blood test | WBC (×10^9^/L) | 13.29 ± 7.04 | 8.61 ± 5.89 | 0.002 |
|  | HB (g/L) | 108.20 ± 21.81 | 104.58 ± 27.21 | 0.530 |
|  | PLT (×10^9^/L) | 205.22 ± 99.66 | 155.44 ± 93.43 | 0.029 |
|  | Neutrophil count (×10^9^/L) | 10.13 (6.87, 16.38) | 6.19 (4.32, 11.16) | 0.005 |
|  | Neutrophil ratio (%) | 91.15 (86.50, 95.12) | 90.10 (78.15, 94.15) | 0.324 |
|  | Lymphocyte count (×10^9^/L) | 0.68 (0.38, 0.94) | 0.49 (0.21, 0.82) | 0.143 |
|  | Lymphocyte ratio (%) | 4.95 (3.45, 7.30) | 6.00 (4.05, 15.60) | 0.175 |
| Renal function | urine output (mL/d) | 1000.00 (550.00, 1200.00) | 1050.00 (410.00, 1750.00) | 0.643 |
|  | Creatinine (ummol/L) | 69.00 (52.00, 123.50) | 66.00 (48.60, 115.00) | 0.544 |
| Liver function | ALT (U/L) | 27.00 (18.25, 42.75) | 18.00 (12.50, 37.50) | 0.137 |
|  | AST (U/L) | 41.00 (21.50, 47.00) | 31.00 (23.00, 49.50) | 0.803 |
|  | TBIL (ummol/L) | 11.70 (7.32, 21.32) | 8.80 (5.65, 12.10) | 0.035 |
|  | Albumin (g/L) | 30.90 (27.55, 36.40) | 28.70 (25.50, 32.15) | 0.055 |
| Inflammatory index | PCT (ng/mL) | 0.62 (0.29, 3.71) | 0.42 (0.16, 1.08) | 0.151 |
|  | CRP (mg/L) |  |  |  |
|  | ESR (mm/h) | 50.00 (33.00, 82.00) | 47.00 (26.00, 81.00) | 0.762 |
| Lymphocyte subsets count | CD3^+^ T cell (/uL) | 469.96 (282.00, 648.52) | 297.54 (126.27, 609.52) | 0.073 |
|  | CD4^+^ T cell (/uL) | 222.69 (160.81, 386.02) | 96.64 (36.50, 280.37) | 0.012 |
|  | CD8^+^ T cell (/uL) | 188.19 (104.02, 287.23) | 159.07 (70.00, 267.00) | 0.299 |
| Cytokine | TNF-α (pg/mL) | 2.15 (1.75, 2.82) | 1.89 (1.09, 2.43) | 0.222 |
|  | IL-6 (pg/mL) | 58.93 (8.11, 128.00) | 28.75 (6.58, 181.50) | 0.867 |
|  | IL-10 (pg/mL) | 2.48 (2.16, 5.30) | 2.98 (2.24, 6.49) | 0.561 |
