# Supplementary material for: Clinical characteristics and risk factors for mortality in pneumonia-associated acute respiratory distress syndrome patients: a single center retrospective cohort study
Source: Front Cell Infect Microbiol. 2024 Jul 9;14:1396088. doi: 10.3389/fcimb.2024.1396088 (PMC11263095; doi:10.3389/fcimb.2024.1396088)
Supplement: Supplementary file 2 [file Table_2.docx]

eTABLE 2 Patients’ outcome

|  |  | **Immunocompetent group**  **(N=39)** | **Immunosuppressive group**  **(N=36)** | ***P* value** |
| --- | --- | --- | --- | --- |
| Primary endpoint | 28-day mortality (%) | 17 (47.22) | 26 (66.67) | 0.089 |
| Secondary endpoint | ICU mortality (%) | 2 (5.56) | 6 (15.38) | 0.316 |
|  | 90d mortality (%) | 18 (50.00) | 28 (71.79) | 0.053 |
|  | Length of ICU (d) | 8.50 (4.75, 12.25) | 9.00 (6.50, 14.00) | 0.221 |
|  | Length of hospital stay (LOS, d) | 12.50 (7.00, 17.00) | 17.00 (8.50, 25.00) | 0.136 |
