# Supplementary material for: Clinical characteristics and risk factors for mortality in pneumonia-associated acute respiratory distress syndrome patients: a single center retrospective cohort study
Source: Front Cell Infect Microbiol. 2024 Jul 9;14:1396088. doi: 10.3389/fcimb.2024.1396088 (PMC11263095; doi:10.3389/fcimb.2024.1396088)
Supplement: Supplementary file 3 [file Table_3.docx]

eTABLE 3 Patients’ outcome in different severity

|  |  | **Mild**  **ARDS**  **(N = 8)** | **Moderate ARDS**  **(N = 36)** | **Severe ARDS**  **(N = 31)** | ***P* value** |
| --- | --- | --- | --- | --- | --- |
| **Primary endpoint** | 28-day mortality (%) | 3(37.5) | 19(52.8) | 21(67.7) | 0.227 |
| **Secondary endpoint** | ICU mortality (%) | 0(0.00) | 5(13.9) | 3(9.7) | 0.502 |
|  | 90d mortality (%) | 3(37.5) | 21(58.3) | 22(71.0) | 0.195 |
|  | Length of ICU (d) | 8(5,9) | 9(6.25,14.5) | 9(3.13) | 0.412 |
|  | Length of hospital stay (LOS, d) | 9(6.25,15) | 15(7.25,19) | 13(9.33) | 0.282 |
